# The Effect of Moxibustion Stimulation on Local and Distal Skin Temperature in Healthy Subjects

**DOI:** 10.1155/2019/3185987

**Published:** 2019-04-02

**Authors:** Ying Li, Chao Sun, Jiujie Kuang, Changchun Ji, Jiangtao Wu

**Affiliations:** ^1^Key Laboratory of Thermo-Fluid Science and Engineering of MOE, Xi'an Jiaotong University, Xi'an 710049, China; ^2^Department of Acupuncture and Moxibustion, Shaanxi Traditional Chinese Medicine Hospital, Xi'an 710003, China; ^3^Bioinspired Engineering and Biomechanics Center (BEBC), Xi'an Jiaotong University, Xi'an 710049, China

## Abstract

The aim of this study is to investigate the response of local and distal skin temperature to moxibustion stimulation (MS) and explore the effects of MS on sympathetic nerve activity. The distal skin temperatures of fingertips, as an indicator for sympathetic reflex response, were recorded using infrared camera during resting period (10 min), MS period (10 min), and natural cooling period (15 min), respectively. The MS without ash cleaning (AC) was applied to acupoints Quze (PC3) (Group I) and Lao Gong (PC8) (Group II), respectively. In Group III, the MS with the operation of AC was performed on PC8. The temperature responses of the local stimulation points and corresponding control points were also investigated. At the beginning of MS period, a significant increase of temperature on the stimulation point accompanied by a simultaneous reduction of temperature on fingertips was observed. A marked negative correlation was also obtained between temperature changes in the stimulation point and in the fingertips. At the end of natural cooling period (*t* = 34 min), the temperature of stimulation point was obviously higher than baseline values. In contrast, the temperatures of fingertips increased and then returned to the baseline levels during the second minute of MS period. In Group III, the temperature of stimulation point increased every time with the operation of AC, accompanied by the temperature decrease of middle fingertip. The findings suggest that moxibustion may trigger the sympathetic nervous system and induce the reduction of microcirculation, accompanied by a reduction of fingertip temperature. In addition, the operation of AC caused repeated cycles of thermal stimulation on the stimulation point, which may repetitively activate cutaneous sympathetic nerve fibres and evoke the temperature reduction of fingertips.

## 1. Introduction

Moxibustion plays an important role in Traditional Chinese Medicine treatments, which treats and prevents diseases by burning a herb preparation containing Moxa (*Artemisia vulgaris*, Mugwort) to stimulate the meridians of human body [1]. Moxibustion has been reported for treating a wide range of diseases, such as breech presentation, ulcerative colitis, fatigue, stroke, and pain [2–5]. Although the exact mechanism of moxibustion is not clear, the thermal effect is considered as a key point for the effectiveness of moxibustion. Thus, the temperature has an essential role during moxibustion [6].

Research has been conducted to investigate the thermal effect of moxibustion on local temperature and blood flow [7–10]. Xu et al. [7] investigated temperature and blood perfusion volume changes on dorsal skin of healthy human subjects and revealed a rapid and sharp increase in both blood perfusion volume and skin temperature after traditional moxibustion treatment. Wang et al. [8] presented that thermal stimulation like moxibustion significantly increased local blood flux and affected heart rate variability. Noguchi et al. [9] found a two-phase response in blood flow, a transient decrease followed by an increase without blood pressure change, when applying moxibustion-like thermal stimulation to the gastrocnemius muscle. Their analysis demonstrated that the increase in blood flow occurred due to an axon reflex that had a reflex arc below the spinal cord, and the transient decrease in blood flow was induced by excitation of postganglionic muscle sympathetic fibres.

Furthermore, several research studies show that the effectiveness of moxibustion is related to the modulation of autonomic nervous system, especially the sympathetic nervous system. The research of Shu et al. [11] showed that moxibustion was more effective than acupuncture in treatment of chronic fatigue syndrome in the long term and the mechanism of this effect may involve both the vagal nerve activation and the modulation of sympathetic nervous system as well. Paterno et al. [12] reported that electroacupuncture and moxibustion had beneficial effects on chronic kidney disease by decreasing the renal sympathetic nerve activity leading to lowered blood pressure. In particular, Matsumoto-Miyazaki et al. [13] evaluated the moxibustion treatment on renal hemodynamic and found a reduction of renal vascular resistance after indirect moxibustion due to autonomic nervous system modulation.

The sympathetic nervous system is a branch of autonomic nervous system and plays a crucial role in the control of the cardiovascular system in humans through the regulation of both cardiac function and peripheral blood flow. Moxibustion has been reported to reduce sympathetic nerve activity in the treatment of chronic kidney disease [12, 13]. However, moxibustion has also been found to excite the activity of muscle sympathetic fibres [9]. Therefore, the effects of moxibustion on sympathetic nervous system activity remain equivocal.

According to the research of Kistler et al. [14], various stimuli (noise, deep breath, and hand cooling) triggering the sympathetic nervous system could induce decreases in cutaneous microcirculation. A transient decrease of fingertip temperature was also observed with a lag phase. Thus, they proposed that fingertip temperature could be considered as an indicator for sympathetic reflex responses and this parameter is easy and simple to analyze. However, very limited literature has been published on fingertip temperature observation under MS. In this study, the skin temperatures of three fingertips (index, middle, and ring finger) and the stimulation point were monitored during MS to determine the effects of moxibustion on both distal and local skin temperature and analyze the response of sympathetic nerve activity to MS. In addition, the changes of skin temperature under different stimulation points and repeated MS were also investigated.

## 2. Materials and Methods

### 2.1. Participants

Thirty-six volunteers, twenty-seven males and nine females, were recruited from the staff and students of Xi'an Jiaotong University in the study. The demography of participants is shown in Table 1. All participants were healthy persons and in good physical condition. No participants had previously undergone moxibustion treatment, and none were taking any medication. Participants were advised to abstain from caffeine and smoking at least 24 hours prior to the experiment. In accordance with the Declaration of Helsinki, all participants provided informed consent and had an adequate understanding of the procedure and purpose of the present study. The study was approved by the Ethics Committee of Xi'an Jiaotong University.

### 2.2. Protocol for Stimulation

**Figure 1:**
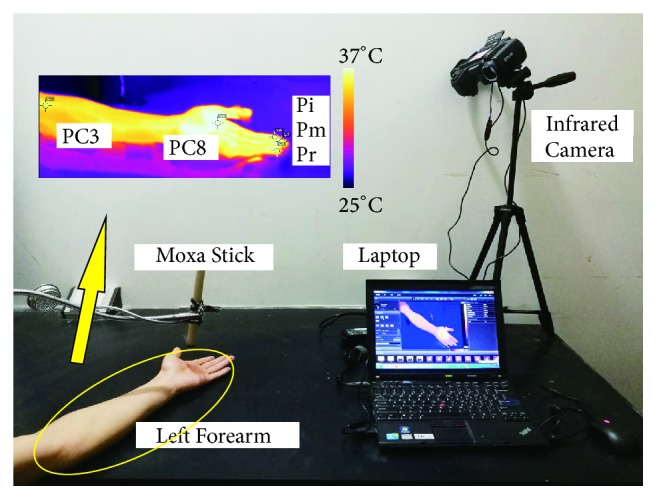
Schematic of the experiment.

In this study, suspended moxibustion was used to carry out experiments, which was performed by holding an ignited moxa stick at a certain distance above the patient's skin surface, keeping the spot warm and making it reddened without burns. Based on the theory of acupuncture and moxibustion in Traditional Chinese Medicine, the Pericardium Meridian is the main vessel of the heart that governs blood circulation in the whole body [15]. The electroacupuncture stimulation on acupoints of the Pericardium Meridian showed a moderating function to improve heart performance [16]. Therefore, the acupoints Quze (PC3) and Lao Gong (PC8) on Pericardium Meridian of Hand-Jueyin were selected as stimulation points. The locations of acupoints PC3 and PC8 are shown in Figure 1. Participants were randomly divided into four groups: MS without AC on PC3 (*n* = 10, denoted as Group I), MS without AC on PC8 (*n* = 10, Group II), MS with AC on PC8 (*n* = 8, Group III), and a Control group (*n* = 8).

The schematic of the experiment and the positions of monitoring points are shown in Figure 1. Experiments were performed in a quiet room at an ambient temperature of 23°C~25°C and humidity of 40%~50%. The participants rested on a chair in the room for 30 minutes before measurements. Before experiments, the left forearms of participants were immobilized on the desk. A calibrated infrared camera (T420, FLIR Systems, Inc.) was adjusted to focus on the left forearm of participants, and the corresponding positions of PC3 and PC8 and three fingertips were labeled on the real-time images as specific locations of pixels using FLIR Tools system, as depicted in Figure 1. During experiments, infrared images of left forearm were recorded continuously throughout the whole experimental process in the computer using FLIR Tools with a sampling rate of 1 Hz. Finally, the temperature data of each monitored point were extracted from infrared images using FLIR Tools. The sensitivity, accuracy, and resolution for the employed camera are 0.045 K, ± 2 K, and 320 × 240 pixels, respectively.

**Figure 2:**
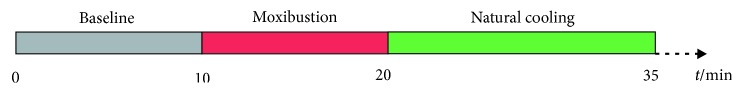
Stimulation schematic diagram.

As shown in Figure 2, the whole experimental duration for three MS groups was 35 min including 10 min of baseline, 10 min of MS period, and 15 min of natural cooling period. For Group I, the moxa stick was ignited and fixed vertically above the skin surface of PC3 at* t* = 10 min. The distance between the bottom surface of moxa sticks and the skin surface was 3 cm, which is the most comfortable distance in the clinical moxibustion treatment [17]. At* t* = 20 min, the burning moxa stick was moved away from skin surface. For Group II, the burning moxa stick was applied 3 cm above the skin surface of PC8. For Group III, during MS period, the burning moxa stick was removed slightly away from above PC8 to clean the ash content on its bottom surface every 3 min (*t* = 13 min, 16 min, 19 min) and then moved back with the same distance of 3 cm between the bottom of moxa sticks and skin surface after every operation of AC. For Control group, participants sat on the chair and the infrared images of left forearms were recorded continuously throughout 35 min including 10 min of baseline, 10 min of MS period with unlit moxa sticks above the skin surface, and 15 min of natural cooling period.

### 2.3. Statistical Analysis

The results are presented as mean values ± standard deviation. Statistical analysis was performed using SPSS 21.0. One-way ANOVA was performed for temperature comparison among groups, and post hoc tests were used for further analysis with significant effects. The paired* t*-test was employed for within-group temperature comparison. The Friedman test was used for temperature comparison among four temperature changes in Group III. Correlations between temperature of the stimulation point and the middle fingertip during moxibustion were analyzed using simple regression and Spearman's rank correlation. Significant differences were indicated by *∗ p* < 0.05 or *∗∗ p* < 0.01.

## 3. Results

### 3.1. MS without AC Applied on PC3 (Group I)

**Figure 3:**
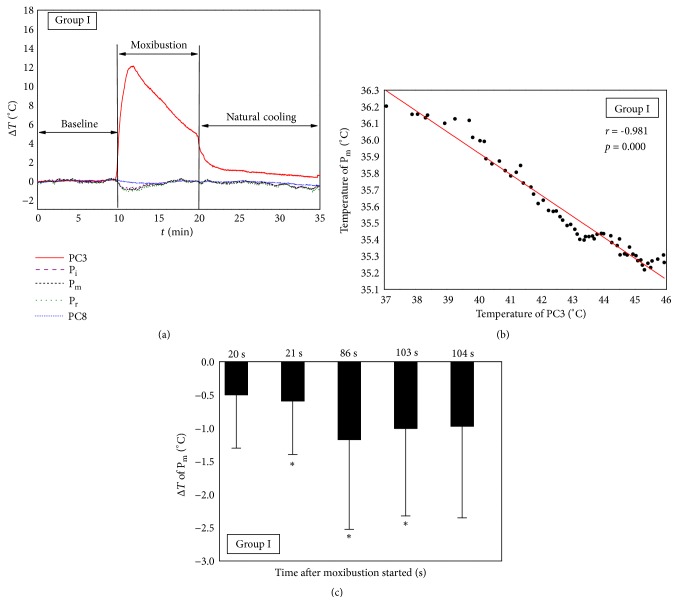
In Group I: (a) mean temperature variation of monitoring points; (b) correlation between temperature of the middle fingertip (P_m_) and stimulation point PC3 during the first minute of MS period; (c) comparison of the temperatures on middle fingertip (P_m_) with baseline values at different times using paired* t*-test. Δ*T* was obtained by subtracting the mean value of baseline from the transient temperature. *∗* represents* p* < 0.05.

The MS was applied on PC3, and the temperature of PC8 was measured as a control point. The temperatures of index fingertip (P_i_), middle fingertip (P_m_), and ring fingertip (P_r_) were monitored to study the response of distal skin temperature to MS on PC3.  Figure 3(a) depicts the mean temperature evolution of monitoring points during MS on PC3 without AC. The temperatures were presented as difference values (Δ*T*) between transient temperatures and the mean values of baselines. As shown in Figure 3(a), at the beginning of MS period, the temperature of PC3 increased immediately from the baseline values. In contrast, the temperatures of three fingertips were significantly decreased after the ignited moxa sticks applied on PC3. The correlation between temperature changes of PC3 and P_m_ during the first minute of MS period was analyzed in Figure 3(b), and a marked negative correlation was observed (*r* = -0.981,* p* < 0.05). As shown in Figure 3(c), statistical analysis was also conducted to compare the temperatures of P_m_ at different times with baseline values using 2-tailed* t*-test for paired data, and the temperature drop was obtained by subtracting the mean value of baseline from the transient temperature of middle fingertip. It can be found that significant temperature drops occurred at the 21 s after moxibustion started and lasted for 82 s. In addition, the temperature of control point PC8 has no obvious change throughout the MS period.

### 3.2. MS without AC Applied on PC8 (Group II)

**Figure 4:**
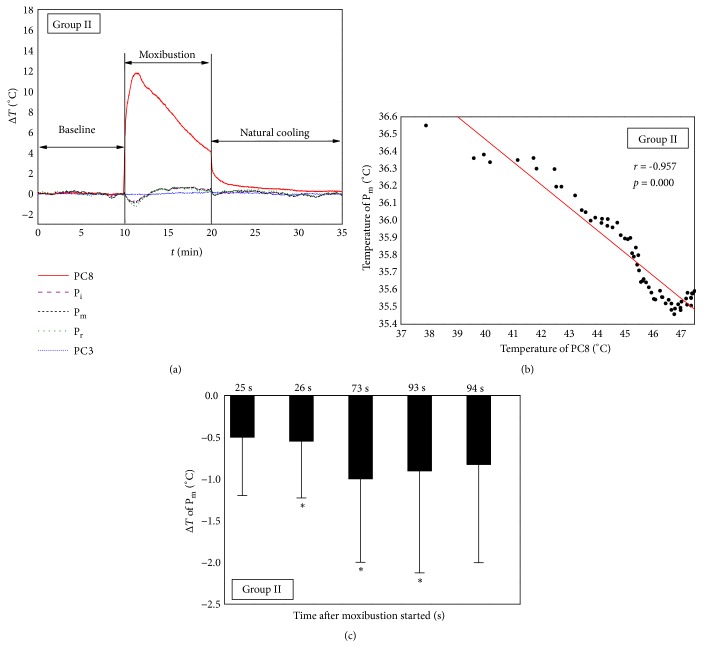
In Group II: (a) mean temperature variation of monitoring points; (b) correlation between temperature of the middle fingertip (P_m_) and stimulation point PC3 during the first minute of MS period; (c) comparison of the temperatures on middle fingertip (P_m_) with baseline values at different times using paired* t*-test. Δ*T* was obtained by subtracting the mean value of baseline from the transient temperature. *∗* represents* p* < 0.05.

The MS was applied on PC8, and the temperature of PC3 was measured as a control point. The temperatures of three fingertips were monitored to study the response of distal skin temperature to MS on PC8.  Figure 4(a) depicts the mean temperature evolution of monitoring points during MS on PC8 without AC. The temperatures were presented as temperature difference values (Δ*T*) between transient temperatures and the mean values of baselines. As shown in Figure 4(a), the temperature of PC8 increased immediately from baseline values when the ignited moxa sticks were applied on PC8. In contrast, the temperatures of three fingertips fell from the baseline values at the beginning of MS. The correlation between temperature changes of PC8 and P_m_ during the first minute of MS period was analyzed in Figure 4(b), and a significant negative correlation was also obtained (*r* = -0.957,* p* < 0.05). The temperatures of P_m_ at different times were compared with baseline values statistically using 2-tailed* t*-test for paired data, and the results were depicted in Figure 4(c). The significant temperature drops occurred at 26 s after moxibustion started and lasted for about 68 s. It can also be seen from Figure 4(a) that the temperature curve of P_r_ was lower than that of P_i_ at the beginning of MS period, although they showed similar variation tendency. Meanwhile, the temperature of control point PC3 had no obvious change throughout the MS period.

### 3.3. MS with AC Applied on PC8 (Group III)

**Figure 5:**
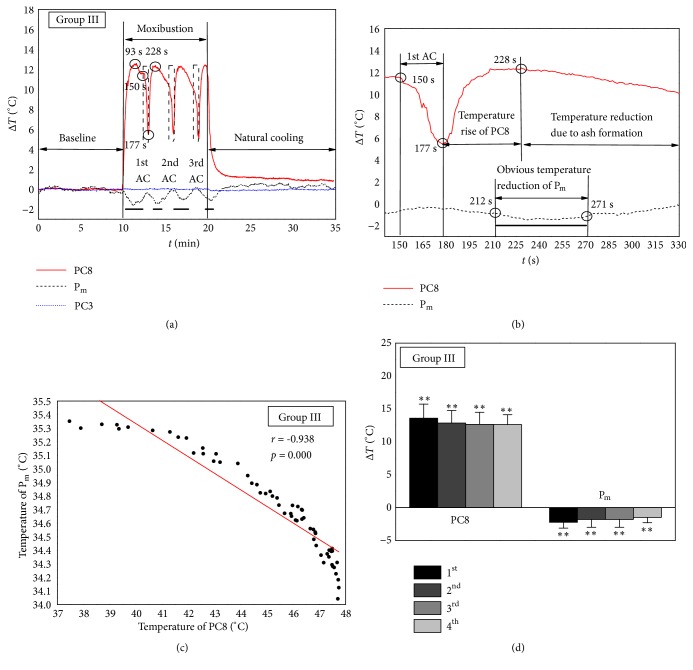
In Group III: (a) mean temperature variation of monitoring points, — represents the time duration of obvious temperature reductions of middle fingertip (P_m_); (b) detailed temperature-time history of stimulation point PC8 and middle fingertip (P_m_) in the first operation of AC (*t* represents the time after MS); (c) correlation between temperature of the middle fingertip (P_m_) and stimulation point PC3 during the first minute of MS period; (d) comparison of the maximum temperatures on stimulation point PC8 at four temperature rises and the minimum temperatures on middle fingertip (P_m_) at four temperature drops with baseline values using paired* t*-test; *∗∗* represents* p *< 0.01.

Figure 5(a) depicts the mean temperature evolution of monitoring points during MS on PC8 with AC. The temperature of PC8 showed a periodic fluctuation with the operation of AC. Correspondingly, the temperature of middle fingertip showed a relevant fluctuation of temperature decrease. At the beginning of MS period, the temperature of PC8 increased with the heat effect of burning moxa sticks and reached the maximum value at 93 s of MS period. Then, the temperature of PC8 decreased gradually because of ash deposition on the surface of burning moxa sticks. Accordingly, the temperature of P_m_ decreased significantly from 16 s of MS period and this obvious temperature reduction lasted to 137 s. The detailed temperature-time history of PC8 and P_m_ in the first operation of AC is shown in Figure 5(b). It can be seen that the temperature of PC8 declined sharply from 150 s to the minimum values due to the removal of burning moxa sticks for AC. After the AC finished, burning moxa sticks were moved back to skin surface of PC8, and the temperature of PC8 increased immediately from 178 s and reached the second maximum value at 228 s. The obvious reduction of P_m_ temperature occurred at 212 s after the temperature rise of PC8 began and lasted to 271 s. The other two operations of AC were performed at 330 s and 510 s of MS period. After each operation of AC, the temperature of PC8 began to increase due to the heat effect of MS, and the obvious temperature reductions of P_m_ occurred with the temperature rise of PC8. These results indicated that the temperature of P_m_ experienced three additional temperature reductions with three repeated increases of PC8 temperature induced by the operation of AC.

The correlation between temperature changes of PC8 and P_m_ was analyzed during the first minute of MS period, and the significant negative correlation (*r* = -0.938,* p* < 0.05) was observed in Figure 5(c). As shown in Figure 5(d), the maximum temperatures of four temperature rises on PC8 and the minimum temperatures of four temperature drops on P_m_ were compared with their baseline values statistically using paired* t*-test, respectively. Also, Friedman test was employed to compare four temperature rises and four temperature drops in Group III, respectively. The results demonstrated that there is no obvious difference among four maximum temperatures on PC8 and among four minimum temperatures on P_m_.

### 3.4. Control Group

**Figure 6:**
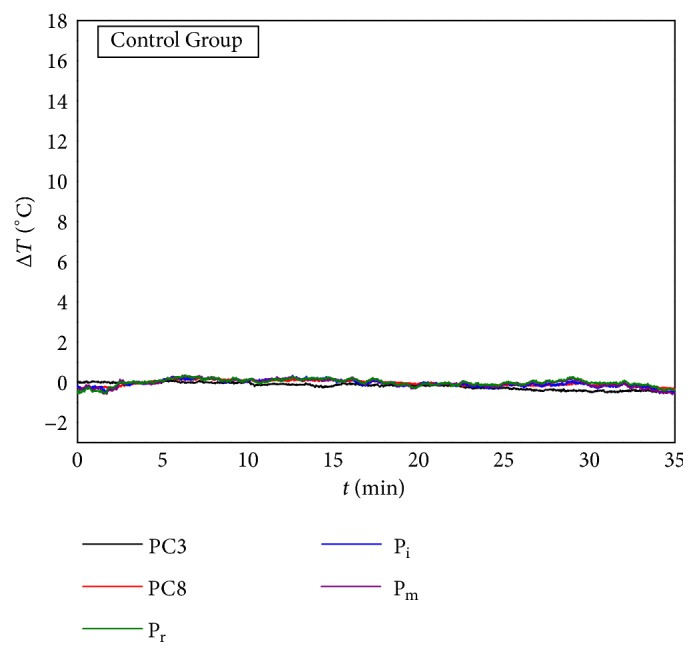
Mean temperature variation of monitoring points in control group.

Figure 6 shows the mean temperature variation of monitoring points in Control group. The first 10 min was considered as baseline period and the second 10 min was set as MS period with unlit moxa sticks above the simulation point, and the last 15 min was set as natural cooling period. The fingertip temperatures fluctuated during 35 min of Control group and the temperature of PC3 was relatively more stable compared with fingertip temperatures, which was consistent with the observation made by Huizenga* et al*. [18]. Considering that the MS-induced temperature changes of fingertips in MS groups occurred mainly in the first 3 minutes of MS period, the temperature changes in the corresponding time period of 10 min to 13 min in Control group were selected for comparison with three MS groups.

**Figure 7:**
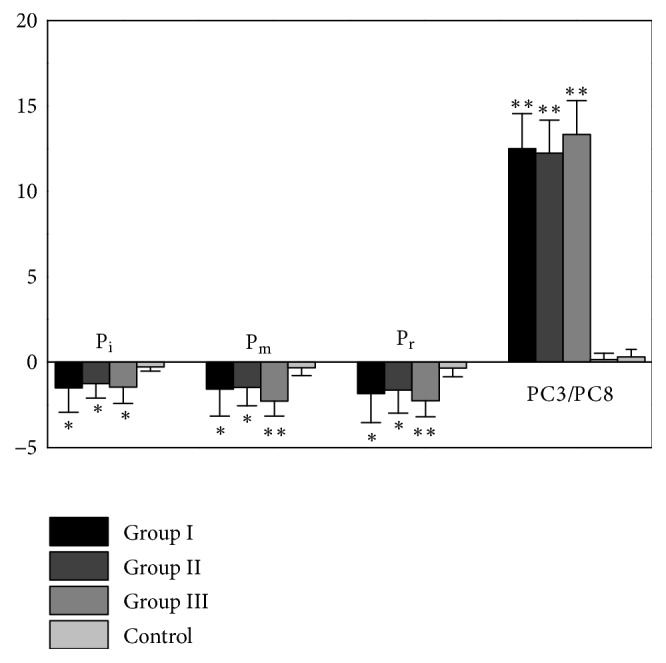
Comparison of the maximum temperature differences of stimulation points (PC3 in Group I, PC8 in Group II and Group III, and PC3 and PC8 in Control group) and three fingertips (P_i_, P_m_, and P_r_) among three MS groups and Control group using one-way ANOVA. *∗* and *∗∗* represent that the values of three MS groups are significantly different from the Control group.

In Figure 7, the maximum temperature rises on stimulation points and the maximum temperature drops on three fingertips at the first 3 minutes of MS period were compared among three MS groups and Control group using ANOVA test with post hoc tests for further analysis. The results demonstrated that temperature changes in stimulation points (PC3 and PC8) and three fingertips in three MS Groups had significant differences with Control group, but no obvious differences were observed among Groups I, II, and III.

## 4. Discussion

### 4.1. Temperature Changes of the Stimulation Point and Fingertips in Responses to MS without AC

In the present study, the temperature evolution on skin surface was recorded during MS. The hand and finger temperature fluctuated significantly when the body was near a neutral thermal state, and the possible explanation is that the body is actively using hand vasodilation and constriction to regulate heat loss to around the neutral conditions [18]. Thus, the fingertip temperature was monitored prior to stimulus and the MS was applied to the forearm when there is a relatively stable baseline. At the beginning of moxibustion, a significant increase of temperature on the stimulation point was observed. According to the research on moxibustion [19, 20], the burning moxa stick produces local temperature increase on the stimulation point. Lin* et al*. [19] measured that the maximum rise of skin surface temperature at SP6 induced by moxibustion was 11°C at 4 min during MS period, which generally agrees with the results of this study (Figure 7). Meanwhile, the temperature rise of stimulation point was accompanied with a simultaneous reduction of temperature on fingertips (Figures 3(a), 4(a), and 5(a)). A marked negative correlation between temperature changes in the stimulation point and fingertips was also obtained (Figures 3(b), 4(b), and 5(c)). The results indicate that moxibustion on the stimulation point results in the temperature decrease of fingertips.

Some research has been reported to study the relation between fingertip temperature decrease and sympathetic nerve activity [14, 21–23]. Kistler* et al.* [14] proposed that the decrease in fingertip temperature was indicative for sympathetic induced changes in microcirculation. They used various stimuli (noise, deep breath, and hand cooling) triggering the sympathetic nervous system to induce decreases in cutaneous microcirculation, with a lag phase of approximately 15 s; the transient decrease of fingertip temperature was observed. In our experiments, the obvious decrease of fingertip temperature was appeared at 21 s in Group I and at 26 s in Group II after the MS was applied (Figures 3(c) and 4(c)). Kenichi Kimura* et al.* [21] also found that, during the first minute of manual acupuncture stimulation, the burst rate of skin sympathetic nerve activity increased, accompanied by a reduction of skin blood flow. As a result, the findings suggested that the moxibustion, as a thermal stimulation, probably could trigger the sympathetic nervous system and induce the reduction of microcirculation, accompanied by a decrease of fingertip temperature.

As mentioned in [3, 24], both acupuncture and moxibustion have effectiveness in treatment of the chronic pain. Reflex sympathetic dystrophy (RSD) is a chronic pain disorder and the affected area of RSD has blood flow rise during the initial stages of the disorder, followed by the blood flow decrease. Landry and Scudds [22] reported that electroacupuncture could produce a short-term cooling of the skin temperature of the hand and finger, which may reduce blood flow, cool the skin in the early stages of RSD, and result in a mitigation in pain. Thus, moxibustion may also have benefit to control the pain in the early stages of RSD. Besides, moxibustion could increase heart rate and mean femoral arterial pressure and have a regulation effect on cardiac function in brachycranial rats with the promotion of the degranulation of mast cells [25]. The degranulation of mast cells may play an important role in signal transmission between mast cells and peripheral sensory nerve [26]. Zhu* et al. *[16] also found that heart rate and blood pressure were improved by electroacupuncture and the mast cell activation in the acupoint PC6 was important for the electroacupuncture effect against pituitrin-induced bradycardia in rabbits. All these findings may suggest that moxibustion stimulation on acupoints of Pericardium Meridian could be beneficial for cardiac function with the regulation of heart rate and blood pressure, and one of the involved underlying mechanisms is related to the trigger of effective signals in neural system.

### 4.2. Comparison of Temperature Changes between Group I and Group II

Group I and Group II were compared to study the effects of stimulation position on fingertips' temperature. Both the temperature rises of stimulation points (PC3 and PC8) and the temperature drop of fingertips were observed in Group I and Group II (Figure 7). The maximum temperature rises on stimulation points and maximum temperature drops on fingertips had no obvious difference between two groups (Figure 7). In Figure 3(c), it can be found that the obvious temperature drops of middle fingertip appeared at 21 s and ended at 103 s of MS period in Group I. Relatively, Figure 4(c) shows that the obvious temperature drops of middle fingertip occurred at 26 s and ended at 93 s of MS period in Group II. These timepoints were important values to characterize the distal temperature changes caused by activation of sympathetic nervous system under moxibustion. In addition, the closer distance between heat source and fingertips in Group II may contribute to the delay occurrence and the shorter duration of obvious fingertip temperature drop.

The temperature on stimulation points decreased after reaching the maximum values in both groups due to ash formation on the surface of burning moxa sticks (Figures 3(a) and 4(a)). However, Δ*T* of stimulation point at the end of MS period (*t *= 20 min) in Group II was smaller than that of Group I (*p* < 0.05) (Figures 3(a) and 4(a)). The results demonstrate that the speed of temperature decline on the stimulation point in Group II is faster than that of Group I, and the reason may be the sweating cooling of the palm, which occurred mainly when the MS was applied on PC8 in Group II. The phenomenon of sweating was also observed during experiments. At the end of natural cooling period (*t* = 34 min), the temperature of PC3 in Figure 3(a) was still significantly higher than baseline values, which indicates that moxibustion has a long-term heat effect on the stimulation point. In contrast, moxibustion elicits a short-term cooling effect on the skin temperature of fingertips, because the temperature of fingertips increased and then returned to the baseline levels during the second minute of MS period in the two groups (Figures 3(c) and 4(c)). Furthermore, the consistent temperature decreases of three fingertips in both groups (Figures 3(a) and 4(a)) suggested that the effect of moxibustion on distal skin temperature was mediated systemically. Correspondingly, Paulson and Shay [23] reported that acupuncture appeared to activate the sympathetic nervous system and caused bilateral decrease in distal skin temperature after needle insertion.

### 4.3. Temperature Changes of the Stimulation Point and Fingertips in Responses to MS with AC

Because the ash deposition on the surface of burning moxa stick has a negative effect on stimulation temperature [27], the operation of AC was performed every 3 min in Group III. With every operation of AC, the temperature of stimulation point increased every time accompanied by the decrease of middle fingertip. Four temperature rises on the stimulation point along with four temperature drops on the fingertip can be seen in Figure 5(a). The results in Figure 5(b) may further indicate that the operation of AC caused the repeated cycles of thermal stimulation on the stimulation point, which may repetitively activate cutaneous sympathetic nerve fibers and evoke the temperature reduction of fingertips.

Repeated stimulation may cause fatigue or sensitization of nervous system [28, 29]. Fatigue refers to the decrement of response with repeated stimulation. Iggo [30] found that the cutaneous mechanoreceptors showed inexcitability because of repeated mechanical stimulus. Sensitization refers to the response increment resulting from novel, strong, or noxious stimulation, which is the main phenomenon in the nociceptive system. Bessou and Perl [31] reported that sensitization of polymodal C nociceptors in the cat was induced by repeated intense heat stimulation (>45°C). Besides, according to clinical research [32], a part of patients reported “sensitized” responses to suspended moxibustion at certain locations on the body, and they felt strong warmth or heat spreading around the stimulating site during treatment. A study of Chen* et al*. [33] indicated that the moxibustion dose in heat-sensitive moxibustion was larger than that in conventional suspended moxibustion. Thus, as an effective approach to strengthen the heat stimulation and produce repeated intense heat stimulation in clinic treatment, the operation of ash cleaning during suspend moxibustion may have advantages for inducing sensitization. Because the occurrence of this heat-sensitization response is often related to obvious better therapeutic effects [34], heat-sensitive moxibustion has been widely used to treat various types of symptoms [35]. Although the heat-sensitization response mainly depends on the selection of the sensitive acupoints associating with pathological state, the operation of ash cleaning, which could induce repeated heat stimulation, may also be a beneficial way to promote the effectiveness of moxibustion.

## 5. Conclusions

In summary, the temperature responses of stimulation points and three fingertips were monitored during MS by means of infrared camera. At the beginning of MS, increased temperature of the stimulation point was accompanied by reduced temperatures of fingertips. The heat effect of MS on the stimulation point lasted until natural cooling period, but the temperatures of fingertips returned to baseline levels during the second minute of MS period. The main findings of the present study imply that MS induces transient activation of skin sympathetic nerves and the repeated cycles of thermal stimulation on the stimulation point caused by operation of AC may repetitively activate cutaneous sympathetic nerve fibers and evoke the temperature reduction of fingertips.

## Figures and Tables

**Table 1:** Demography of participants in experiments (27M/9F).

	Age [years]	Height [cm]	Weight [kg]	BMI
Min	21	154	45	18.97
Max	34	180	90	27.78
Mean ± SD	25 ± 3	169.11 ± 7.20	61.64 ± 9.21	21.45 ± 1.92

## Data Availability

The data used to support the findings of this study are available from the corresponding author upon request.

## References

[1] World Health Organization Western Pacific Region (2007). *WHO International Standard Terminologies on Traditional Medicine in the Western Pacific Region*.

[2] Huang Y., Ma Z., Cui Y. (2017). Effects of herb-partitioned moxibustion on the miRNA expression profiles in colon from rats with DSS-induced ulcerative colitis. *Evidence-Based Complementary and Alternative Medicine*.

[3] Yang M., Chen X., Bo L. (2017). Moxibustion for pain relief in patients with primary dysmenorrhea: A randomized controlled trial. *PLoS ONE*.

[4] Liu C., Chen J., Chang X. (2017). Comparative metabolomics study on therapeutic mechanism of electro-acupuncture and moxibustion on rats with chronic atrophic gastritis (CAG). *Scientific Reports*.

[5] Liu N., Jiang Y., Xing M. (2018). Digital gene expression profiling analysis of aged mice under moxibustion treatment. *Evidence-Based Complementary and Alternative Medicine*.

[6] Deng H., Shen X. (2013). The mechanism of moxibustion: ancient theory and modern research. *Evidence-Based Complementary and Alternative Medicine*.

[7] Xu Q., Yang J., Wang L. (2014). Use of laser speckle contrast imaging to reveal changes in temperature and blood perfusion in the skin of healthy subjects after administration of heated moxa sticks and Daiwenjiu ointment. *Journal of Traditional Chinese Medicine*.

[8] Wang G., Jia S., Li H. (2018). Exploring the relationship between blood flux signals and hrv following different thermal stimulations using complexity analysis. *Scientific Reports*.

[9] Noguchi E., Ohsawa H., Takagi K. (2009). Neural mechanism of localized changes in skeletal muscle blood flow caused by moxibustion-like thermal stimulation of anesthetized rats. *The Journal of Physiological Sciences*.

[10] Wang G., Jia S., Li H. (2017). Changes of blood flux at BL21 and points along BL meridian resulted from acupuncture or moxibustion: group cross design study. *Evidence-Based Complementary and Alternative Medicine*.

[11] Shu Q., Wang H., Litscher D. (2016). Acupuncture and moxibustion have different effects on fatigue by regulating the autonomic nervous system: a pilot controlled clinical trial. *Scientific Reports*.

[12] Paterno J. C., Bergamaschi C. T., Campos R. R. (2012). Electroacupuncture and moxibustion decrease renal sympathetic nerve activity and retard progression of renal disease in rats. *Kidney and Blood Pressure Research*.

[13] Matsumoto-Miyazaki J., Miyazaki N., Murata I. (2016). Traditional thermal therapy with indirect moxibustion decreases renal arterial resistive index in patients with chronic kidney disease. *The Journal of Alternative and Complementary Medicine*.

[14] Kistler A., Mariauzouls C., von Berlepsch K. (1998). Fingertip temperature as an indicator for sympathetic responses. *International Journal of Psychophysiology*.

[15] Zhang C., Wen Y., Fan X.-N. (2015). Therapeutic effects of different durations of acupuncture on rats with middle cerebral artery occlusion. *Neural Regeneration Research*.

[16] Zhu H., Wang X., Huang M. (2017). Mast cell activation in the acupoint is important for the electroacupuncture effect against pituitrin-induced bradycardia in rabbits. *Scientific Reports*.

[17] Liu Q., Sun T., Liang H. (2017). Clinical observation on the correlation between moxibustion sensation and distance of moxa stick. *Journal of Acupuncture and Tuina Science*.

[18] Huizenga C., Zhang H., Arens E., Wang D. (2004). Skin and core temperature response to partial- and whole-body heating and cooling. *Journal of Thermal Biology*.

[19] Lin L.-M., Wang S.-F., Lee R.-P. (2013). Changes in skin surface temperature at an acupuncture point with moxibustion. *Acupuncture in Medicine*.

[20] Li Y., Sun C., Kuang J. (2018). An in vitro and numerical study of moxibustion therapy on biological tissue. *IEEE Transactions on Biomedical Engineering*.

[21] Kimura K., Masuda K., Wakayama I. (2006). Changes in skin blood flow and skin sympathetic nerve activity in response to manual acupuncture stimulation in humans. *American Journal of Chinese Medicine*.

[22] Landry M. D., Scudds R. A. (1996). The cooling effects of electroacupuncture on the skin temperature of the hand. *Journal of Hand Therapy*.

[23] Paulson K. L., Shay B. L. (2013). Sympathetic nervous system responses to acupuncture and non-penetrating sham acupuncture in experimental forearm pain: a single-blind randomised descriptive study. *Acupuncture in Medicine*.

[24] White A., Foster N. E., Cummings M., Barlas P. (2007). Acupuncture treatment for chronic knee pain: a systematic review. *Rheumatology*.

[25] Wang Y. S., Zhang J. B., Jiang J. F. (2013). Research on effects of the thermal stimulation by moxibustion at different temperatures on cardiac function in rats and on mast cells in the local site of moxibustion. *Evidence-Based Complementary and Alternative Medicine*.

[26] Sa Z.-Y., Huang M., Zhang D., Ding G.-H. (2013). Relationship between regional mast cell activity and peripheral nerve discharges during manual acupuncture stimulation of "Zusanli" (ST 36). *Acupuncture Research*.

[27] Sun C., Li Y., Kuang J., Ji C., Wu J. The effect of ash cleaning cycles on thermal characteristics of moxibustion therapy.

[28] Peng Y. B., Ringkamp M., Meyer R. A., Campbell J. N. (2003). Fatigue and paradoxical enhancement of heat response in C-fiber nociceptors from cross-modal excitation. *The Journal of Neuroscience*.

[29] Prescott S. A. (1998). Interactions between depression and facilitation within neural networks: updating the dual-process theory of plasticity. *Learning & Memory*.

[30] Iggo A. (1960). Cutaneous mechanoreceptors with afferent C fibres. *The Journal of Physiology*.

[31] Bessou P., Perl E. R. (1969). Response of cutaneous sensory units with unmyelinated fibers to noxious stimuli. *Journal of Neurophysiology*.

[32] Wang J., Yi M., Zhang C. (2015). Cortical activities of heat-sensitization responses in suspended moxibustion: an EEG source analysis with sLORETA. *Cognitive Neurodynamics*.

[33] Chen R., Chen M., Xiong J. (2012). Is there difference between the effects of two-dose stimulation for knee osteoarthritis in the treatment of heat-sensitive moxibustion. *Evidence-Based Complementary and Alternative Medicine*.

[34] Chen R., Xiong J., Chi Z., Zhang B. (2012). Heat-sensitive moxibustion for lumbar disc herniation: a meta-analysis of randomized controlled trials. *Journal of Traditional Chinese Medicine*.

[35] Chen R., Chen M., Xiong J., Yi F., Chi Z., Zhang B. (2010). Comparison of heat-sensitive moxibustion versus fluticasone/salmeterol (seretide) combination in the treatment of chronic persistent asthma: design of a multicenter randomized controlled trial. *Trials*.

